# High intensity ultrasound-assisted quality enhancing of the marinated egg: Gel properties and *in vitro* digestion analysis

**DOI:** 10.1016/j.ultsonch.2022.106036

**Published:** 2022-05-18

**Authors:** Zhihui Yu, Huirong Zhang, Haoran Guo, Lixin Zhang, Xiaoyu Zhang, Yisheng Chen

**Affiliations:** a College of Food Science and Engineering, Shanxi Agricultural University, Taigu 030801, Shanxi, China; b Insitute of Food Nutrition and Safety, Shanxi Agricultural University, Taiyuan, 030031, Shanxi, China; c Shanxi Key Laboratory of Edible Fungi for Loess Plateau, Taigu 030801, Shanxi, China

**Keywords:** High intensity ultrasonication, Marinated egg, Gel properties, *In vitro* digestibility

## Abstract

In this study, high intensity ultrasonication (HIU) was employed as an efficient tool to improve the gel property and *in vitro* digestibility of marinated egg (ME). The effects of HIU treatment at 100 W and 200 W for a series of time periods (0.5 h, 1 h, and 2 h) on the textural profiles, structural changes, and microstructures were also studied. After HIU treatment, the springiness and gumminess of ME white were enhanced. The water holding capacity reached the highest point (66.6%) when 0.5 h 200 W HIU was used. It was observed that 100 W HIU led to the highest zeta potential (-12.0 mV) and hydrophobicity (175.35 μg) of ME, indicating a high degree of electrostatic repulsion prevented agglomeration. HIU treatment at 100 W affected the dynamic rheological behaviors by boosting non-covalent bonds, which maintains the gel network's homogeneity. Meanwhile, the decreasing formation of α-helix, in contrast to β-turn, altered the aggregation behaviors of egg white gel. The microstructure of the 200 W HIU treated samples had porous colloidal network structures, and the *in vitro* digestibility (>75%) was increased after HIU. This work demonstrated that HIU could be a green and cost-effective tool for processing the egg product with high quality.

## Highlights

• HIU treatment enhanced the springiness and gumminess of marinated egg.
• The zeta potential and hydrophobicity of protein gels improve after HIU.
• HIU at 100 W affects rheological behaviors by boosting non-covalent bonds.
• The aggregation behaviors are due to the decrease in α-helix while the increase in β-turn.
• The porous network structure and *in vitro* digestibility were also enhanced after HIU.

## Graphical abstract

**Figure:**
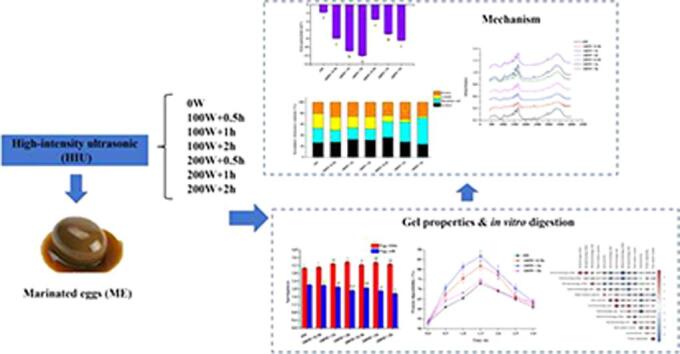

## 1 Introduction

The marinated egg (ME) is a traditional egg product with a long history, popular in many Asian countries [1]. The processing of ME mainly includes physical and chemical changes such as thermal denaturation of proteins, flavoring and coloring. ME gel is a reticulated protein structure induced by heat, of which properties are essentially dependent on the aggregation of denatured proteins. During the processing, the solubility and foaming capabilities of egg protein disappear, while the egg white gel forms, exhibiting enhanced flexibility and gelability, and consequently better taste binding ability [2].

The quality of ME is strongly influenced by key factors such as ionic strength, heat treatment techniques, and temperature. These conditions impact the structure and characteristics of protein gel by altering the route of protein aggregation. So far, there have been several studies aiming to disclose the mechanism of ME gel. For example, Ma et al. [3], [4] reported that certain soluble ovalbumin (OVA) aggregates became insoluble precipitates as the shape of OVA changed from long chains to a more dense network during drying at 75 °C. As a result, the degree of unfolding of protein molecules during pre-gelation influenced the characteristics of protein gel. Egg white proteins are fully unfolded before aggregation, and the hydrophobic groups are exposed to increase intermolecular gravitational forces, resulting in strong aggregates. In addition, the ionic strength and main components of the brine also have effects on the egg white gel strength of ME. The addition of salt and sugar increased the hardness and springiness of protein gel, while pH alterations reduced their mechanical characteristics and water holding capacity (WHC). Pickling time also improved the protein gel mesh structure with increased free sulfhydryl content while dropping total sulfhydryl level during pickling [5]. Additionally, tea and illicium verum were capable of improving egg white gel strength and free sulfhydryl groups in boiled egg [6]. Therefore, changes in external conditions or treatment of substances during the braising process have variable effects on the gel properties of ME.

In recent years, ultrasound-assisted treatment as a green and cost-efficient technology for extracting proteins and improving their functional properties attracted marked attention. Especially, it was reported that the use of high intensity ultrasound (HIU) treatment increased soy protein aggregates with a high relative molecular mass as well as altered microenvironments for aromatic and aliphatic side chains [7]. In addition to improving the secondary structure of soy protein, HIU pretreatment also promotes the formation of less compact tertiary conformation. Moreover, HIU treatment can also effectively enhance protein digestibility [2]. Similar results showed that the combined heat and HIU treatment increased whey protein retention, facilitated a looser microstructure, and decreased hardness [8]. Furthermore, it was evidenced that HIU treatment was also associated with molecular chain rearrangement, structural degradation, and increased digestibility [9]. Therefore, ultrasound technology may open a new horizon for improving the quality of ME.

This work investigated the effects of HIU treatment at different power and time levels on egg white gel and gastrointestinal digestion properties of ME. More specifically, the effects of HIU on the protein gel properties, including texture profile, water loss, zeta potential, hydrophobicity, sulfhydryl content, and rheological properties, *in vitro* digestion were analyzed. Fourier transform infrared spectroscopy (FT-IR), ultraviolet–visible analysis (UV), and scanning electron microscopy (SEM) were also performed to discloasestructural changes.

## 2 Materials and methods

### 2.1 Materials

Fresh eggs (50–60 g) were purchased from HomeLife Supermarket (Taigu, Jinzhong, Shanxi). Salt, sugar, soy sauce, fennel, illicium verum, cinnamon, allspice, ginger, angelica, dahurica, peppercorns, sannai, and other spices were bought from Jiajiali Shopping Supermarket (Taigu, Jinzhong, Shanxi). Sodium chloride (NaCl), potassium dihydrogen phosphate (KH_2_PO_4_), sodium hydroxide (NaOH), and bromophenol blue were obtained from Tianjin Damao Chemical Reagent Co. Ltd. (Tianjin, China). Bio-Bioengineering Co., Ltd. (Shanghai, China) provided the BCA protein assay kit. Spectral grade potassium bromide (KBr) was supplied from Aladdin Chemistry Co. (Shanghai, China). Main enzymes such as pepsins and trypsins and bile salts were purchased from Shanghai Yuanye Bio-Technology Co., Ltd (Shanghai, China).

### 2.2 Sample preparation

Forty-two eggs (50–60 g) were washed, boiled in water for 8 min, and then soaked in cold water for 3 min and shelled manually. The brine solution was prepared with boiling water containing 1.5% salt, 2% sugar, 2% soy sauce, 0.4% fennel, 0.4% illicium verum, 0.4% cinnamon, 0.4% allspice, 0.2% ginger, 0.2% angelica, 0.2% dahurica, 0.2% pepper, and 0.2% sanai [2]. The shelled eggs were boiled in brine for 15 min in a ratio of 100 mL of brine water per egg. The eggs cooked in brine solution were randomly divided into seven groups (6 eggs per group). The pH of brine solution was adjusted to 6.8 in the early pickling stage. HIU experiments were performed in an ultrasonic bath (KQ5200DE, Kunshan Ultrasonic Instruments Co., Ltd., Kunshan, China) for a series of time periods (0.5 h, 1 h, and 2 h) at different powers. The HIU frequencies were set at 40 kHz and power levels were 100 and 200 W, respectively. In a 1000-mL laboratory beaker, five eggs were gently submerged in brine solution (500 mL). The marinating liquid during whole HIU process was kept at 25 °C. After HIU treatment, it was slightly cooled, sealed with plastic wrap, and soaked. After cooling, the eggs were immersed in the brine solution for a total of eight hours (including HIU treatment and marination time).

### 2.3 Texture profile analysis (TPA)

The texture properties such as hardness, springiness, and chewiness of ME were analyzed using a texture analyzer (TMS-PRO, Food Technology Corporation, USA). The whole eggs were divided into two halves along the center line and the egg white and yolk were separated. The yolk was cut into round slices (1 cm) and six spots in the central areas were selected as measurement sites. Six points at the top, bottom and center of the remaining egg white halves were selected. In brief, the selected egg white and yolk were separated from ME and placed individually in plastic molds (20 cm × 15 cm × 15 cm). The test speed was set as 120 mm/min. The interval between cyclic compression tests was set to 2 s, and the trigger force was set to 0.4 N. For egg whites and yolks, the target downward displacement was set to 15 mm and 3 mm, respectively. There were six replicates per group, each containing three eggs.

### 2.4 Color measurement

The color of ME was determined using a Minolta CR-400 colorimeter (Minolta Co. Ltd., Japan). All measurements were performed on the surface area of the ME white (1.5 mm). The color evaluation procedure was based on the determination of values L* (lightness/brightness), a* (redness/greenness), and b* (yellowness/blueness). The color values were expressed as average of three measurements [10]. The whiteness was calculated using equation as follows:

$$\mathrm{Whiteness}\;\left(WH\right)=100-[{\left(100-L^{*}\right)}^{2}+{a^{*}}^{2}+{b^{*}}^{2}]$$

### 2.5 WHC

Four grams of egg white gel with a size of 2 mm × 1 mm × 1 mm were centrifuged (4000 r/min, 10 min, 25 °C). The egg white gel was weighed using a FA2004 electronic balance (Sedolis Scientific Instruments Co., Ltd., Beijing, China), after removing excess water with absorbent paper. The WHC was calculated as follows (%):

$$\mathrm{WHC}\left(\%\right)=\frac{m1}{m0}\times 100$$

where *m*_0_ is the original egg white gel weight, and *m*_1_ is the egg white gel weight after centrifugation.

### 2.6 Zeta potential measurement

The ME white samples were freeze-dried using a lyophilizer (ZG-20, Hangzhou Creative Equipment Co., Ltd., Hangzhou, China). The protein samples were diluted with distilled water until the final protein concentration was 1%. Five mL of solution was filtered through a membrane filter (diameter, 13 mm; pore size, 0.45 μm) (Sartorius, Gottingen, Germany) and diluted twice with PBS buffer (0.01 g/L, pH 7.4). The Nano-ZS90 (Malvern Instruments Ltd., UK) by placing the sample (0.75 mL) in the high concentration cell equipped with a transparent electrode was used to determine the zeta potential of ME white samples [11].

### 2.7 Surface hydrophobicity analysis

The hydrophobicity of ME white was measured using the method of Chen et al. [12]. The protein sample of 1 mL with a concentration of 1% was mixed with 200 μL of bromophenol blue (BPB, 1 mg/mL), and then stirred at room temperature for 10 min. The supernatant was collected after centrifugation (2000 rpm for 15 min at 4 °C) and added to a 9-fold PBS buffer. The absorption value (A) was measured at 595 nm using a UV–visible spectrophotometer (WFJT200, Shanghai Mepro Delta Instruments Co., Ltd., Shanghai). The blank control (A0) was determined at 595 nm against a control solution without the sample. The following equation was used to assess surface hydrophobicity:

$$\mathrm{BPB}\;bound\;\left(\mu g\right)=\frac{A_{0}-A}{A_{0}}\times 100$$

### 2.8 Free sulfhydryl (SHF) and total sulfhydryl (SHT)

The SHT and SHF were determined by the Ellman method [13]. The SHF group content levels of egg white solution samples were determined by diluting 1 mL of the solution (4 mg/mL) with Tris-Gly buffer (10.18 g Tris, 6.76 g Glycine, and 1.49 g EDTA dissolved in 1 L of distilled water, pH 8.0). After centrifugation for 15 min at 12,000 g at 4 °C, 3 mL of the supernatant were mixed with 50 μL of Ellman's reagent (10 mM 5,5′-dithiobis 2-nitrobenzoic acid (DTNB) in Tris-Gly buffer). The reaction was incubated at 25 °C for 1 h, protected from light. The absorbance was measured at 412 nm against a blank of reagent. The same procedure was applied to determine the SHT group content. For the SHT content, the protein sample solution (1 mg/mL) was diluted with Tris-Gly buff that contained 8 M urea and 0.5% sodium dodecyl sulfate (w/v).

### 2.9 Determination of rheological behavior

The rheology of ME white samples was measured using a rheometer (DHR-1, TA Instruments Inc., New Castle, DE, USA). The samples were placed on a 40 mm aluminum parallel plate with a measurement gap of 1 mm. The linear viscoelastic behavior of the sample is determined at frequency scan by a sweep strain of 0.01–100% at a frequency of 1 Hz. Throughout the procedure, the storage modulus (G′) and loss modulus (G″) were continuously recorded.

### 2.10 UV spectral scanning

One mL of egg white solution samples (4 mg/mL) was diluted 10-fold with PBS (10 mM, pH 7.0). The protein solution was determined with an ultraviolet spectrophotometer (UV1901PC, Shanghai Aoxiang Scientific Instrument Co., Ltd.). The spectra were recorded between 200 and 400 nm with a scanning speed of 5 S/Nm.

### 2.11 FT-IR analysis

One mg of dried ME white sample was mixed with dry spectroscopic grade potassium bromide powder (1 g) and pressed into tablets with a diameter of 1 mm. The FT-IR scans were conducted from 400 cm^−1^ to 4000 cm^−1^ using a spectrometer (Thermo, Waltham, MA, USA), and the data was processed using OMNIC version 8.0 software.

### 2.12 SEM

The change in morphology of egg protein powder after desalting was examined by scanning electron microscope (Hitachi S-800, Hitachi Co. Ltd., Tokyo, Japan). After fixing with 2.5% glutaraldehyde for 2 h, the samples were washed with distilled water for 5 min. After dehydrating in a graded ethanol series for 30 min (five steps of 10% increments, starting with 60% ethanol), the samples were observed using a gaseous secondary electron detector at a voltage of 10 kV.

### 2.13 *In vitro* gastrointestinal digestion

The gastrointestinal digestion experiments *in vitro* were performed according to the method of Gu and Wu [14] with some modifications. For further study, the collected ME were air-dried and milled through a 40-mesh sieve. The digestion of egg protein powder was simulated by dissolving it in 20 mL of hydrochloric acid (pH 1.5) and mixing it with 15 mg of pepsin (1200 U/g). The mixture was adjusted to a pH of 3.0 ± 0.3 with 1 M HCl and then 37 °C water bath for 3 h to facilitate gastric digestion. Pepsinolysis was terminated by adjusting the pH to 7.0 with 1 M NaOH. The digest was then mixed with 20 mL of digest containing bile salts (10 mmol/L) and 100 U/mL of trypsin. Following that, the above combination was incubated at 37 °C for 4 h with continuous shaking (140 r/min). To terminate the digestion, the mixture was boiled for 5 min to inactivate the enzyme, followed by 30 min of cooling in an ice bath. The supernatant was collected after centrifugation at 8000 r/min for 30 min at 4 °C. The soluble protein concentration in the supernatant was determined by the Pierce bicinchoninic acid (BCA) protein assay kit (Thermo Fisher Scientific). To determine the total protein content of the egg, approximately 0.5 g of egg protein was added to the Automatic Kieldahl Apparatus (K9860, Hanon Instruments, Jinan, China). The *in vitro* protein digestibility was calculated as follows:

$$\text{Protein}\;\text{Digestibility(\%)}=\frac{\text{Protein}\;\text{in}\;\text{supernatant}}{\text{Total}\;\text{protein}\;\text{content}}\times 100$$

### 2.14 Statistical analysis

All experiments were performed three times. Analysis of variance (ANOVA) was performed using SPSS Statistics 22 software, with a *P* < 0.05 indicating a significant difference, and a *P* > 0.05 indicating a nonsignificant difference, and plotted with origin 8.5.

## 3 Results and discussion

### 3.1 Effect of HIU treatment on the TPA properties and color of ME

**Fig. 1:**
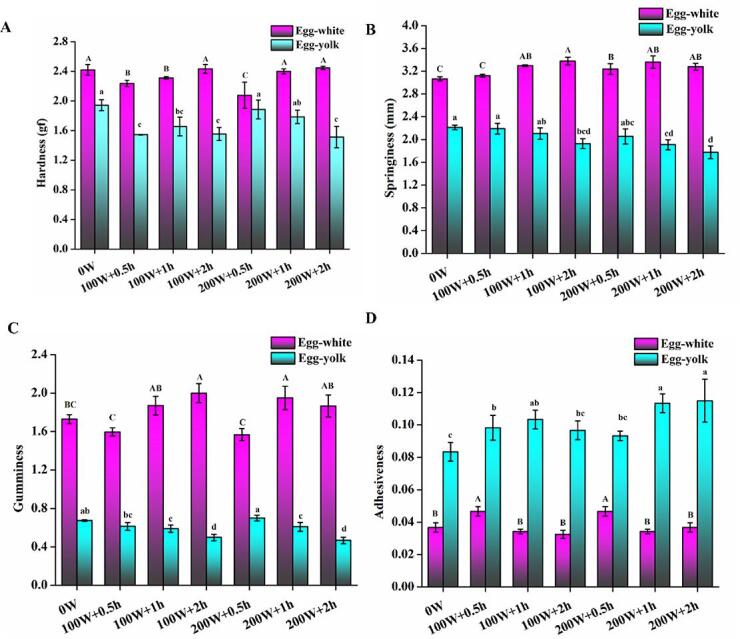
Effect of HIU treatment on the textural profiles of ME. A, Hardness; B, Springiness; C, Gumminess; D, Adhesiveness. The data are expressed as Mean ± SD, n = 3; The different lower-case letters (a–d) above the same column indicate statistically significant differences at *P* < 0.05.

Among the TPA properties of ME white gel, hardness and springiness serve as significant indicators of gel-forming ability [15]. As shown in Fig. 1A, ME whites treated with HIU for 0.5 h had a significant reduction in hardness (*P* < 0.05), whereas those treated for 2 h had no significant effect on hardness as compared with the control group (*P* > 0.05). The gel hardness of egg yolk was drastically reduced after 100 W HIU treatment than the control group (*P* < 0.05). It was reported that the gel network structure may be disrupted by the high temperature generated by ultrasound. Furthermore, an increase in moisture content may result in a decrease in hardness [2]. The springiness of egg white gel after HIU treatment was significantly enhanced than that of the control group (0 W) (*P* < 0.05). As the ultrasound time was extended, the springiness of the egg yolk gradually decreased. Importantly, the springiness of egg yolk was considerably reduced under HIU treatment for 2 h compared to those in the control group (*P* < 0.05) (Fig. 1B). The level of white gumminess increased with the increase of time and reached its peak at 2.0 with 100 W + 2 h treatment (Fig. 1C). A decreasing trend in egg white adhesiveness was observed with time and the lowest value (0.03) was obtained at 100 W + 2 h (Fig. 1D).

Egg white adhesiveness exhibited a similar pattern to the gel gumminess of egg yolk. In the 200 W + 2 h group, the egg yolk had the highest adhesiveness of 0.115. Meanwhile, HIU treatment at 100 W for 0.5 h significantly improved the adhesiveness of egg yolk (*P* < 0.05), while this effect was not significant (*P* > 0.05) with longer treatment duration. This implies that the duration and power of HIU increased the gel strength of egg proteins. Xie et al. [16] observed similar results that sonication improved the springiness and cohesiveness of egg yolk gel. Ultrasound has been shown in several studies to enhance protein-protein and protein-water interactions, resulting in tighter association and improved gel properties of proteins. Therefore, it was concluded that the textural properties of ME gel are related to the formation of reticular structures after thermal coagulation, which was facilitated to a great extent by HIU treatment [13].

Compared with the 0 W group, HIU had no significant effect on the brightness (L*) and WH values (P > 0.05) (Table 1). The values of a* significantly increased after HIU treatment for 0.5 h and 1 h, and the highest a* was observed in 200 W + 0.5 h (*P* < 0.05). After HIU treatment, the b* value of ME increased significantly, while the power and time had no significant effects on it. This indicates that the a* and b* of ME white increased after ultrasound treatment. It was reported that HIU accelerated Na^+^ penetration into the egg white, while the yolk membrane and lipids prevent the infiltration of Na^+^ into the egg yolk [17]. The penetration of Na^+^ is also accompanied by the penetration of soy sauce, spices, and salt in the marinade solution. The cavitation effect of ultrasound could form more homogeneous and stable spatial network structures of ME white, which promote the mass transfer process and accelerates the penetration of the marinade. Na^+^ infiltration also caused structural changes in low-density lipoprotein (LDL) in egg yolk. LDL contains most of the lipids in egg yolk, and free lipids are released from LDL micelles due to structural changes in LDL caused by high temperature and Na^+^ infiltration [18]. However, the cavitation of HIU at 200 W leads to structural aggregation of egg whites and further inhibits the entry of Na^+^, leading to a decrease in a* values.

**Table 1:** Effect of HIU treatment on the color of ME.

Group	L*	a*	b*	WH
0 W	44.48 ± 2.77^a^	9.77 ± 0.82^c^	21.32 ± 0.38^b^	39.96 ± 2.01^a^
100 W + 0.5 h	45.30 ± 3.29^a^	11.55 ± 0.73^b^	25.12 ± 1.90^a^	40.55 ± 1.75^a^
100 W + 1 h	45.89 ± 2.69^a^	12.75 ± 0.55^ab^	25.48 ± 0.60^a^	38.93 ± 2.67^a^
100 W + 2 h	46.87 ± 0.17^a^	9.99 ± 0.56^c^	24.74 ± 1.60^a^	41.25 ± 0.48^a^
200 W + 0.5 h	44.65 ± 1.57^a^	13.82 ± 1.04^a^	24.12 ± 0.46^a^	33.63 ± 0.44^ab^
200 W + 1 h	47.62 ± 1.33^a^	10.04 ± 0.78^c^	26.00 ± 1.28^a^	40.11 ± 1.47^a^
200 W + 2 h	46.14 ± 1.57^a^	9.46 ± 0.04^c^	26.09 ± 0.41^a^	40.43 ± 0.54^a^

### 3.2 Effect of HIU treatment on WHC and hydrophobicity of ME

**Fig. 2:**
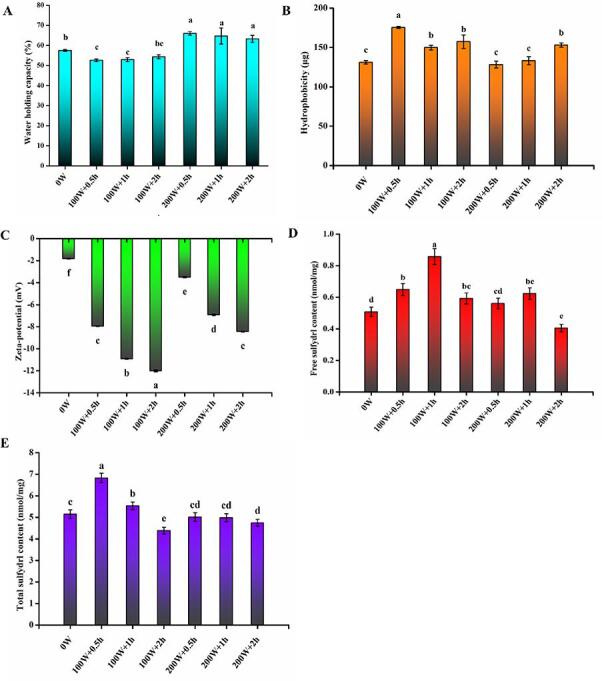
Effect of HIU treatment on the gel properties and surface properties of ME. A, WHC; B, Hydrophobicity; C, zeta-potential; D, free sulfhydryl content; E, total sulfhydryl content. The data are expressed as Mean ± SD, n = 3; The different lower-case letters (a–c) above the same column indicate statistically significant differences at *P* < 0.05.

The WHC has been used as an important indicator to visualize water retention based on self-structuring [19]. The WHC in the 100 W + 0.5 h and 100 W + 1 h groups was significantly lower than that of the control group (*P* < 0.05). Additionally, a slight but not statistically significant increase was observed in the HIU-treated groups for 2 h (*P* > 0.05) (Fig. 2A). This may be due to the fact that the HIU with lower power and shorter duration may break the hydrogen bonds in the secondary structure of the protein gel, which results in lower WHC of gel [20]. The WHC of egg white gel in the 200 W + 0.5 h group was the highest, reaching a value of 66.03%. It dropped slightly at 200 W with the extension of treatment time, and was much higher than that of the HIU treated at 100 W (*P* < 0.05). This may be explained by an increase in gel strength. Higher gel strength created denser networks, which improve WHC by trapping large volumes of water in the gel. In addition, the HIU treatment may cause more protein chains to unfold and clump together, which strengthens the gel structure and makes the gel more water-soluble [21]. Therefore, the results indicated that HIU at higher power caused some de-folding and aggregation of proteins in ME white gel, resulting in more homogeneous and stable spatial network structures with stronger binding to water molecules [16]. Also, the higher WHC was in agreement with the higher egg white gel adhesiveness that was found in the textural analysis.

As shown in Fig. 2B, the hydrophobicity was markedly increased in HIU treated at 100 W compared to the control group, reaching a maximum of 175.35 μg in the 100 W + 0.5 h group. However, the surface hydrophobicity of ME protein decreased significantly in 100 W + 1 h (*P* < 0.05). Hydrophobicity did not differ significantly between the 200 W + 0.5 h and control groups (*P* > 0.05). In contrast, the hydrophobicity in the 200 W + 2 h group was significantly increased than that of the control group (*P* < 0.05). These results show that the change in hydrophobicity was strongly correlated with protein gelation. A recent study has shown that HIU treatment could cause partial expansion of polypeptide chains inside proteins and exposure of hydrophobic groups, consistent with our results [22]. The cavitation effect created by HIU treatment may cause changes in folding between the protein gel molecules combined with hydrophobic exposure. These findings also suggest that HIU treatment at 200 W may promote the formation of covalent and hydrogen bonds, which can reduce protein hydrophobic interactions and increase protein gel strength.

### 3.3 Effect of HIU treatment on the zeta potential and sulfhydryl groups of ME

The zeta potential analysis showed a negative zeta potential of −12.0 mV to −1.82 mV for all groups, and the absolute value of the zeta potential was significantly higher after treatment with HIU (*P* < 0.05). The absolute zeta potential values increased with time at the same ultrasound power (Fig. 2C). The highest zeta potential for ME (-12.0 mV) was found in the 100 W + 2 h group, indicating a high degree of electrostatic repulsion prevents agglomeration. In contrast, the absolute values of zeta potentials in HIU treated at 200 W groups were significantly lower than those in HIU treated at 100 W groups. Particle aggregation and dispersion in ME are mostly determined by their effective surface charges [23]. The higher zeta potential with HIU treatment at 100 W might be explained by more exposure of polar groups in the protein, which was consistent with the previous study [13]. HIU treatment at 100 W could also strengthen electrostatic repulsion between soybean protein isolate (SPI) particles, rupture existing SPI aggregates, and reduce future aggregate formation [24].

The effects of HIU treatment on the SHF content and SHT content of ME are shown in Fig. 2D and Fig. 2E, respectively. After 100 W HIU treatment, the SHF content increased significantly compared to the control group (*P* < 0.05). While the lowest levels of SHF were found in the 200 W + 2 h group (0.41 nmol/mg). As seen in Fig. 2E, the SHT content increased significantly in the 100 W + 0.5 h and 100 W + 1 h groups, while decreased sharply in the 100 W + 2 h group (*P* < 0.05). However, the SHT content did not change significantly at the different time points under 200 W HIU treatment (*P* > 0.05). This indicates that the majority of sulfhydryl residues within the protein molecules in ME were subjected to denaturation after 100 W HIU treatment. In contrast, prolonged HIU treatment generated the extra folded and intramolecular disulfide bonds through oxidation of SH, which eventually resulted in a reduction in the SHT content  in the proteins [15].

### 3.4 Effect of HIU treatment on the rheological properties of ME

**Fig. 3:**
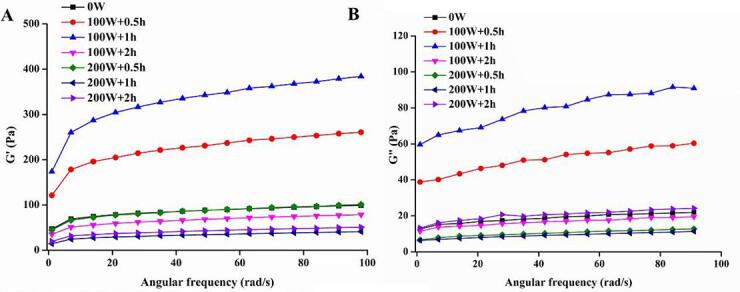
Effect of HIU treatment on the rheological properties of ME. A, storage modulus (G′); B, loss modulus (G″).

Fig. 3 illustrates the effect of various HIU treatments on the changes of G′ and G″ of ME protein gel. The G′ may be used to evaluate the elasticity and resilience of gel samples. A higher G′ implies stronger cross-links and better structure. The G″ reflects the dissipation of viscous energy throughout the process [25]. The G′ and G″ of all samples increases as the angular frequency increases. Furthermore, the G′ of each group is higher than the corresponding G″. The G″ values in the HIU treatment groups at 100 W + 0.5 h and 100 W + 1 h were higher than that in the control group throughout measurement. This demonstrates that the ultrasonic treatment led to greater aggregation of ME proteins by increasing their hydrophobicity. Protein aggregates are mostly composed of covalent bonds and non-covalent bonds [26]. Particularly, disulfide bonds exhibited a distinct influence on the network structure and mechanical strength of the gel, promoting the formation of more stable gels. This suggests that lower power ultrasonic treatment caused protein unfolding and enhanced connections between the protein molecules in ME, presumably by boosting non-covalent bonds, such as hydrogen bonds, which contributes to the stable gel network structure [27]. Researchers found that the springiness of the samples was larger than their adhesiveness, indicating the nature of viscoelastic solids [28]. The results in egg white gel springiness are consistent with those in frequency sweeps, which shows that there is a favorable relationship between rheological and textural qualities [29].

### 3.5 Effect of HIU on the structural properties of ME white gel

**Fig. 4:**
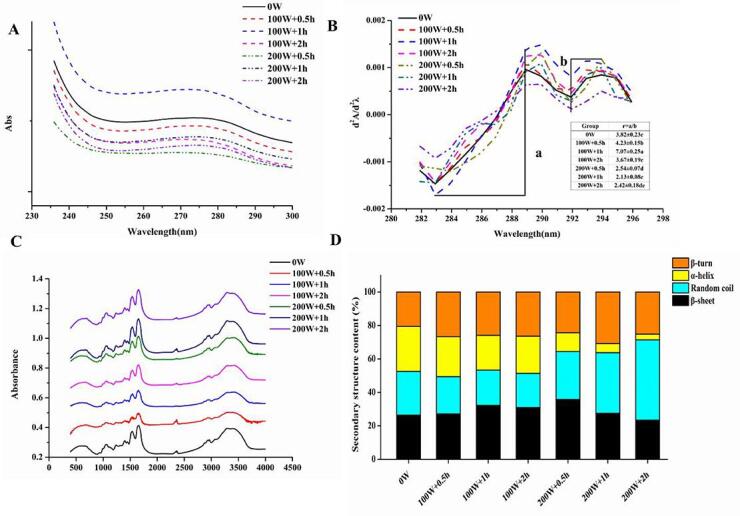
Effect of HIU treatment on the structures of ME. A, UV first derivative; B, UV second derivative; C, FTIR; D, secondary structures content.

The changes in UV absorption spectra can indicate changes in amino acid environment, and protein structure [30]. As shown in Fig. 4A, the highest absorbance value was observed in the 100 W + 1 h group, which is in agreement with rheological results. In the second derivative spectrum (Fig. 4B), the peak at 289 nm was attributed to the synergistic effect of tyrosine (Tyr) and tryptophan (Trp) residues, while the peak at 294 nm represents the spectral characteristics of tryptophan [31]. Compared to the control group (0 W), the peak of tryptophan in each ultrasound group has a significant redshift, which indicates the high polarity of the solvent environment. The ratio r = a/b of the distance between two peaks and troughs was calculated to determine the microenvironmental changes of tyrosine residues [32]. The r value (7.07) increased significantly in 100 W + 1 h than that in the control group (3.82) (*P* < 0.05). The r value was observed to reach its maximum in 100 W + 1 h, followed by a downward trend. The r values were considerably lower in the 200 W treated groups than those in the control group. The r value increased in 100 W + 1 h, indicating tyrosine residues have unfolded and been exposed to polarity. Similar results showed that ultrasound-assisted heating treatment improved the emulsification properties of 11S globulin by increasing the r value. This shows that HIU can induce the migration of more Tyr residues to the nonpolar region by changing the protein structure [33]. The decrease in the r value by HIU treatment with higher power due to the enhanced hydrophobic interactions and reassembly of protein molecules [22].

Fig. 4C illustrates the FT-IR spectrum of ME white gel treated with HIU. The spectral region between 1600 and 1700 cm^−1^ ascribed to the amide I vibration of proteins, which is connected to the stretching vibration of the C = O molecule. The peak intensities in the 3750–3100 cm^−1^ region (N–H stretching and hydrogen bonding) of ME white gel were significantly different with various power levels, which affects the hydration capacity of egg proteins [34]. At the same time, the characteristic absorption peaks (amide I band) were observed in all groups, whereas the amide I band showed a general peak intensity decrease in 100 W treated groups without a shifted peak position compared to the control group. The second derivatives of the amide I band of samples were determined and fitted using the Gauss area method. The results indicated that the components of protein secondary structures were affected by the ultrasound power and treatment time (Fig. 4D).

Four types of secondary structures (β-turn, α-helix, random coil, and β-sheet) in the control group were distributed relatively evenly, and α-helix accounted for the largest proportion of total structure (26.88%). It was found that the α-helix was the most stable protein secondary structure, and that its relative content decreased substantially (*P* < 0.05) when HIU power and HIU duration increased. As a result, the stability of ME white gel structure decreases, which results in the gradual breakdown of hydrogen bonds in the α-helix formation. The protein aggregates further affect the stability of ME white gel structure. A negative correlation has been found between the α-helix and egg white gel hardness, and reductions in the α-helix attenuate the hydrogen bond strength, further leading to a breakdown of the molecular structure of ME white gel [35]. Compared to the control group, HIU treatment resulted in a considerable increase in the content of β-turn, while treatment duration had no effect on the content of β-turn. Additionally, the content of the random coil decreased with increased treatment time at 100 W but showed the opposite effect at 200 W. This trend is associated with the denaturation of ME white gel and the formation of aggregates [36]. Thus, it can be concluded that the β-sheet and β-turn structures formed during HIU treatments contribute to the aggregation behaviors of ME white gel.

### 3.6 Effect of HIU treatment on the microstructure of ME

**Fig. 5:**
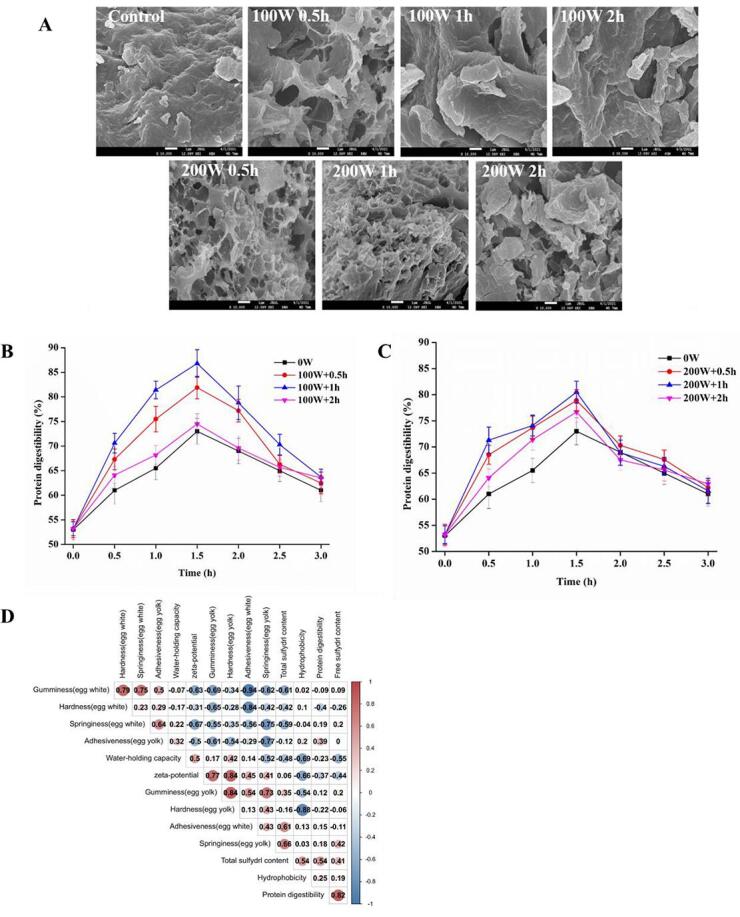
Effect of HIU treatment on the microstructures and *in vitro* digestibility of ME. A, SEM images; B *in vitro* digestibility under HIU at 100 W; C, *in vitro* digestibility under HIU at 200 W; D, correlation analysis.

The effects of ultrasound on the properties of ME protein gel were further investigated by evaluating surface morphology, and the results are shown in Fig. 5A. The surface of the protein gel without HIU treatment is unevenly lamellar and relatively flat and dense, which indicates an ordered network structure due to the stretching of the protein [37]. The HIU treated ME exhibited some fragmentation on the egg white gel surface compared to the control group. Particularly, cracks or depressions were observed on some spherical particles with increased roughness after the HIU treatment at 100 W. The microstructure of the 200 W HIU treated samples had porous colloidal network structures, and the pore loading structure was more uniform under 0.5 h and 1 h treatments. SEM of egg white protein revealed more fragments and irregular holes with increased ultrasonic power. However, after an increase in the sonication time, the pore-loaded structures clearly aggregated into larger clumps, indicating an accelerated aggregation process. These structural features imply that ME proteins are more likely to form pore-like structures under high power sonication [38].

### 3.7 Effect of HIU on *in vitro* digestibility of ME white gel

Protein digestibility can be used to determine the rate and extent of protein digestion in food protein and is one of the most critical attributes to determine the nutritional characteristics of food [39]. Proteins with a high digestibility rate are more likely to be digested and absorbed by the body. As shown in Fig. 5B and C. The digestibility of all groups increased first and then declined, reaching a maximum at 1.5 h. The digestibility dramatically depends on the microstructure of food, as the stable network structure reduces the digestibility. The digestibility did not reach 85% after 1 h of digestion, probably owing to the stable network structure of ME white. Moreover, the digestibility of ME was effectively improved in all HIU groups, with the highest digestibility (86.8%) in 100 W + 1 h at 1.5 h of digestion. The improved digestibility after HIU may be attributed to the fact that moderate HIU can stretch the structure of egg protein and increase the binding sites of digestive enzymes, thus improving the digestibility of egg protein [40]. Additionally, it is suggested that cavitation enhances the *in vitro* digestibility of ME proteins after HIU by increasing the combination of molecules in the brine solution. This enhances the thermal stability of proteins and reduces the effect of excessive heating on the structure of ME proteins. Previous study also revealed that high temperature destroyed the gel structure and improved the digestibility of boiled eggs. However, sustained high temperature induced the protein aggregation, and the reactions of proteins with sugar or lipids, would reduce the digestibility [41]. Thus, the proteins inside ME will not be excessively damaged by heat, leading to increased *in vitro* digestibility [2].

### 3.8 Correlation analysis

An analysis of the relationship between the gel structure, structural parameters, and the *in vitro* digestion was conducted using Pearson correlation (Fig. 5D). The correlation coefficients for gumminess (egg yolk) and hardness (egg yolk) are 0.77 (*P* < 0.05) or 0.84 (*P* < 0.05), respectively. The study found that egg yolk gumminess and hardness were positively related to zeta potential. The correlation coefficient between hydrophobicity and WHC was −0.69 (*P* < 0.05), and hydrophobicity and hardness correlated negatively in egg yolk (0.88). It was found that the correlation between WHC and hydrophobicity was significant, and a decrease in hydrophobicity increased both WHC and gel strength in egg yolk. Notably, the correlation coefficients between protein digestibility and SHF content were 0.82 (*P* < 0.05). HIU enhances digestibility by increasing the exposure of SHF and generating more disulfide bonds. Higher hydrophobicity could lead to an increase in the hydrophobic interaction between the protease and the interfacial protein [39]. Additionally, it has been demonstrated that disulfide bonds, hydrophobic interactions, and secondary structure of proteins can influence their *in vitro* digestibility [40].

## 4 Conclusion

The gel properties of ME were strongly impacted by HIU treatment. HIU treatment effectively improved the texture profiles and color properties by increasing the springiness and a* of egg white gel, which is related to intermolecular force changes in heat-induced gel. For egg white gel, HIU at 200 W for 2 h enhanced the WHC and hydrophobicity. In addition, HIU treatment at 100 W for 1 h caused partial unfolding of the protein structure and surface exposure of the sulfhydryl group, as shown by the increase in zeta-potential or SHF content. More porous micellar network structures were observed in HIU at 200 W. The cavitation and microturbulence caused a progressive breakdown of hydrogen bonds in the α-helix and higher digestibility. This paper presents the gel properties, structural characteristics, and *in vitro* digestibility of ME were significantly improved by HIU treatment. This work develops a novel technology for ME preparation and expands the use of HIU in food processing.

### CRediT authorship contribution statement

**Zhihui Yu:** Conceptualization, Writing – original draft. **Huirong Zhang:** Methodology, Writing – original draft. **Haoran Guo:** Writing – original draft. **Lixin Zhang:** Writing – original draft. **Xiaoyu Zhang:** Conceptualization. **Yisheng Chen:** Funding acquisition, Conceptualization, Writing – original draft.

## Declaration of Competing Interest

The authors declare that they have no known competing financial interests or personal relationships that could have appeared to influence the work reported in this paper.
